# Exploring the shared gene signatures of smoking-related osteoporosis and chronic obstructive pulmonary disease using machine learning algorithms

**DOI:** 10.3389/fmolb.2023.1204031

**Published:** 2023-05-11

**Authors:** Haotian Wang, Shaoshuo Li, Baixing Chen, Mao Wu, Heng Yin, Yang Shao, Jianwei Wang

**Affiliations:** ^1^ Graduate School of Nanjing University of Chinese Medicine, Nanjing, China; ^2^ Department of Traumatology and Orthopedics, Wuxi Affiliated Hospital of Nanjing University of Chinese Medicine, Wuxi, China; ^3^ Department of Development and Regeneration, University of Leuven, Leuven, Belgium

**Keywords:** cigarette smoking, osteoporosis, chronic obstructive pulmonary disease, bioinformatic analysis, machine learning algorithm, immune infiltration

## Abstract

**Objectives:** Cigarette smoking has been recognized as a predisposing factor for both osteoporosis (OP) and chronic obstructive pulmonary disease (COPD). This study aimed to investigate the shared gene signatures affected by cigarette smoking in OP and COPD through gene expression profiling.

**Materials and methods:** Microarray datasets (GSE11784, GSE13850, GSE10006, and GSE103174) were obtained from Gene Expression Omnibus (GEO) and analyzed for differentially expressed genes (DEGs) and weighted gene co-expression network analysis (WGCNA). Least absolute shrinkage and selection operator (LASSO) regression method and a random forest (RF) machine learning algorithm were used to identify candidate biomarkers. The diagnostic value of the method was assessed using logistic regression and receiver operating characteristic (ROC) curve analysis. Finally, immune cell infiltration was analyzed to identify dysregulated immune cells in cigarette smoking-induced COPD.

**Results:** In the smoking-related OP and COPD datasets, 2858 and 280 DEGs were identified, respectively. WGCNA revealed 982 genes strongly correlated with smoking-related OP, of which 32 overlapped with the hub genes of COPD. Gene Ontology (GO) enrichment analysis showed that the overlapping genes were enriched in the immune system category. Using LASSO regression and RF machine learning, six candidate genes were identified, and a logistic regression model was constructed, which had high diagnostic values for both the training set and external validation datasets. The area under the curves (AUCs) were 0.83 and 0.99, respectively. Immune cell infiltration analysis revealed dysregulation in several immune cells, and six immune-associated genes were identified for smoking-related OP and COPD, namely, mucosa-associated lymphoid tissue lymphoma translocation protein 1 (MALT1), tissue-type plasminogen activator (PLAT), sodium channel 1 subunit alpha (SCNN1A), sine oculis homeobox 3 (SIX3), sperm-associated antigen 9 (SPAG9), and vacuolar protein sorting 35 (VPS35).

**Conclusion:** The findings suggest that immune cell infiltration profiles play a significant role in the shared pathogenesis of smoking-related OP and COPD. The results could provide valuable insights for developing novel therapeutic strategies for managing these disorders, as well as shedding light on their pathogenesis.

## 1 Introduction

Persistent airflow llimitation is a distincitve time-developing symbol of COPD, a common preventable and treatable disease (Lippi et al., 2022). It has been found that there is a causation between enhanced chronic inflammatory and COPD, especially with a plus of the effect of cigarette smoking (Brightling and Greening, 2019). COPD is regarded as a systemic disease that is accompanied byseveral comorbid conditions, such as lung cancer, muscle wasting, diabetes, atherosclerosis, orthostatic hypotension, and anxiety/depression. (Romme et al., 2015; Sarkar et al., 2015). The clinical management of these comorbidities is crucial owing to their high correlation to the rates of hospitalization, mortality, and reduced health-related quality of life are commonly observed in patients with COPD (Frei et al., 2014).

Osteoporosis, a systemic bone disease, is considered as one of the significant comorbidities associated with COPD (Kanis et al., 2008). Recent epidemiological evidence suggests a high prevalence of OP in individuals with COPD, despite the absence of a clearly established causal or molecular link between the two disorders (Graat-Verboom et al., 2009; Lehouck et al., 2011; Regan and Jaramillo, 2012; Watanabe et al., 2015). Retrospective chart reviews of 234 male patients with OP from a single bone clinic revealed that COPD accounted for the leading cause of secondary OP (Ryan et al., 2011). Smoking, however, has been found to be a risk factor for both OP and COPD, according to recent studies (Ward and Klesges, 2001; Kanis et al., 2005; Hikichi et al., 2019; Li et al., 2022). Smoking has been shown to cause changes in the microstructure of trabecular bones, as well as reduce the resistance of skeletal muscles to mechanical stress and loading. (Brook et al., 2012). Studies have provided evidence of a functional interplay between bone and immune cells, particularly involving activated T cells and Th17 cells (Kong et al., 1999; Sato et al., 2006; Chen et al., 2016). Additionally, interleukin-17 A expression has been demonstrated to increase in the airways of patients with COPD and correlates with decreased lung function in these patients (Di Stefano et al., 2009; Eustace et al., 2011). In light of these findings, IL-17 A could serve as a common mechanism linking smoking-related OP and COPD. (Xiong et al., 2020).

The occurrence of OP in patients with COPD is asymptomatic and is often undiagnosed until the occurrence of bone fractures. Notably, fractures resulting from OP can further impair the pulmonary function of patients with COPD. Thus, the interdependence between COPD and OP gives rise to a deleterious cycle that imposes a considerable burden on affected individuals. As the occurrence of OP in patients with COPD is immensely underrated, it is essential to elucidate the pathogenesis of OP in COPD, and the early diagnosis of patients with COPD who are at a high risk of OP should be emphasized. The rapid progress in high-throughput microarray technologies has enabled the identification of putative novel biomarkers, genetic variations, and biological pathways, thereby enhancing our comprehension of the pathogenesis and therapeutic intervention of various ailments (Li et al., 2021; Zhou Y et al., 2022). WGCNA (Langfelder and Horvath, 2008; Abu-Jamous and Clust, 2018; Zhou Z et al., 2022) and machine learning techniques are gradually improving, and these bioinformatics tools can be employed for providing great prospects for identifying the potential molecular mechanisms, biomarkers, and therapeutic targets for smoking-related OP and COPD, as well as other complex diseases. These technologies enable researchers to explore large datasets and identify key molecular pathways and biomarkers, leading to the discovery of new therapeutic interventions and improved patient outcomes (Kumar et al., 2021; Chen et al., 2022).

As far as we are aware, there have been few investigations targeting the identification of immune-related biomarkers for the diagnosis of smoking-related OP and COPD and there is a scarcity of studies on the application of machine learning approaches for the diagnosis of these diseases. Smoking-related OP and COPD dataset was obtained from the GEO database and used for the identification of candidate genes in this study. In this study, we employed machine learning algorithms to identify feature genes from candidate genes and validated them using ROC curves. Our study identified potential immune-related diagnostic biomarkers for smoking-related OP in COPD patients, which could facilitate the early diagnosis and personalized treatment of COPD. Our work not only provides a approach for identifying potential biomarkers for COPD but also highlights the value of machine learning algorithms in identifying key genes for complex diseases. The findings of this study have potential implications for improving the clinical management and outcome of COPD patients, and may also provide a framework for the development of similar approaches for other complex diseases.

## 2 Materials and methods

### 2.1 Microarray data

**FIGURE 1:**
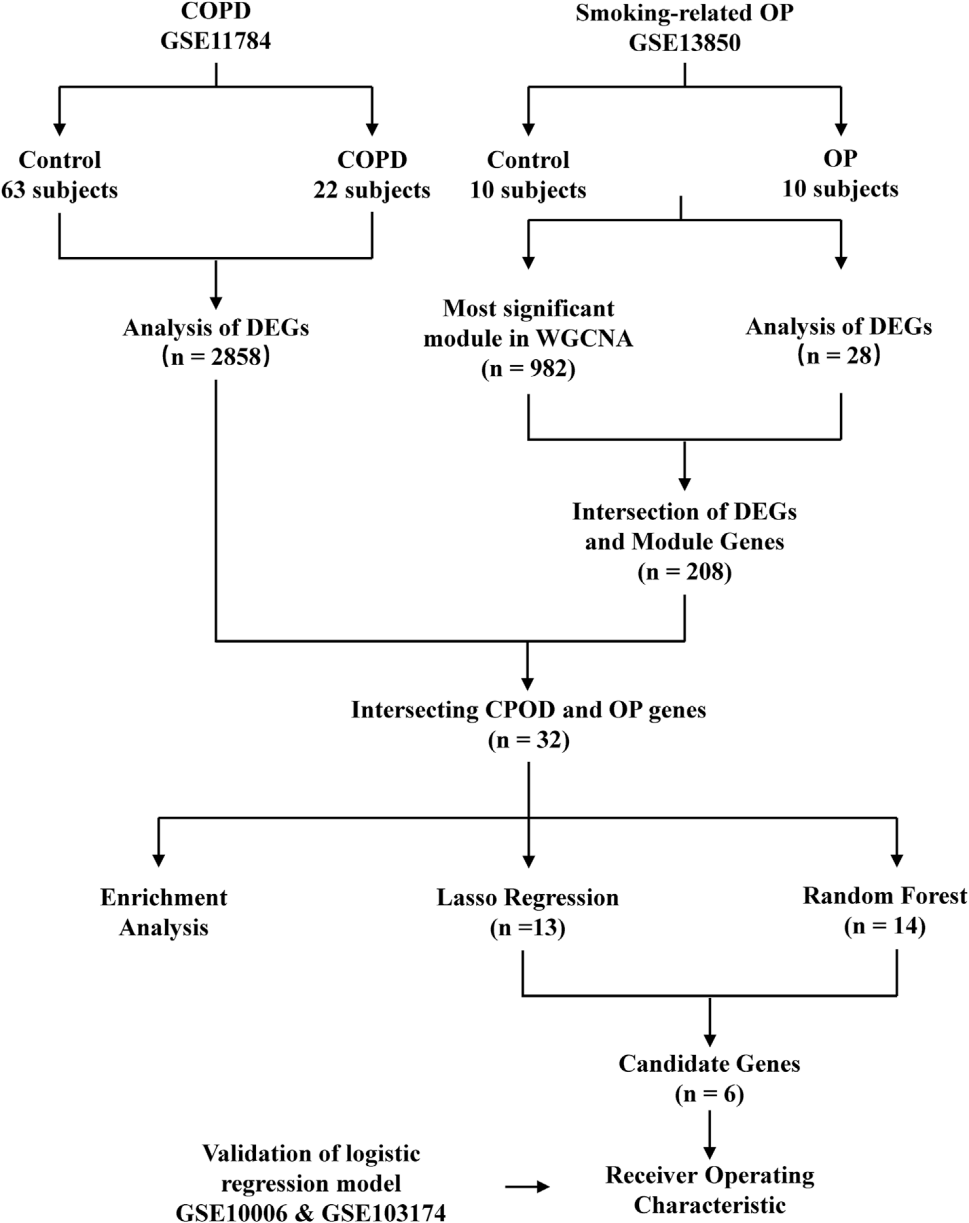
Flowchart depicting the study design. GSE, GEO series; DEGs, differentially expressed genes; OP, osteoporosis.

The smoking-related OP and COPD datasets were retrieved from the GEO database (https://www.ncbi.nlm.nih.gov/geo/) (Edgar et al., 2002) for analyzing the gene expression data in smoking-related OP and COPD. The search strategy employed in this study is described hereafter. The gene expression profiles generated by array analyses were initially retrieved from the GEO database. The OP and COPD datasets, containing samples obtained from blood and the small airway epithelium, respectively, were retrieved from the GEO database. Datasets containing samples of control groups and samples corresponding to *Homo sapiens* were also retrieved from the GEO database. The GSE11784 dataset (Tilley et al., 2011), generated using the GPL570 [HG-U133_Plus_2] Affymetrix Human Genome U133 Plus 2.0 Array platform, contained the expression data of 22 smokers with COPD and 63 healthy subjects. The GSE13850 dataset, also generated using the GPL96 [HG-U133 A] Affymetrix Human Genome U133 A Array platform, contained the expression data of 10 smokers with low bone mineral density (BMD) and 10 healthy subjects. Two validation datasets were subsequently retrieved from the GEO database; one dataset was extracted from the GSE10006 dataset (Carolan et al., 1950), also generated using the GPL570 array platform, and contained the expression data of 54 smokers with COPD and 22 healthy subjects. The other validation dataset was extracted from the GSE103174 dataset and contained the gene expression data of 10 healthy subjects and 23 smokers with COPD. The study did not require the approval of an ethics committee or informed consent as these data are publicly available (Figure 1).

### 2.2 Data processing and analysis of differential gene expression

The DEGs were identified using the limma package in R (version 4.1.1; http://cran.r-project.org/) (Ritchie et al., 2015), and subsequently analyzed using different packages. The initial normalization of the data was performed by applying the normalizeBetweenArrays function from the limma package in R. Subsequently, probe IDs were transformed into gene symbols. The lmFit and eBayes functions were used to calculate the adjusted *p*-values and |log2 (fold change)| of the genes, respectively. DEGs were identified based on the criteria of |log2 (fold change)| > 1.5 and adjusted *p*-value < 0.05.

### 2.3 WGCNA and selection of module genes

The WGCNA system biology strategy was used to investigate the gene-gene correlations using the WGCNA package in R (version 4.1.1; http://cran.r-project.org/), (Langfelder and Horvath, 2008). The gene expression profiles were subjected to Median Absolute Deviation (MAD) calculation for each gene. The calculation steps for MAD are as follows: first, calculate the median of the dataset, and then take the median of the absolute deviation of each data point to obtain the value of MAD. To eliminate the top 30% genes with the smallest MAD, and to remove outlier genes and samples, the goodSamplesGenes function in the WGCNA package of R was utilized. Subsequently, a scale-free co-expression network was constructed using the WGCNA package. Initially, Pearson’s correlation matrices were established to examine all pairwise gene relationships, with the average linkage approach. The correlation strength between gene pairs was used to build a weighted adjacency matrix through the following power function: A_mn = |C_mn|^β; where, C_mn represents the Pearson’s correlation between genes m and n, A_mn represents the adjacency between genes m and n, and *β* is a soft-thresholding parameter that emphasizes strong gene correlations while downplaying weak ones. We set *β* to 4 and converted the adjacency matrix to a topological overlap matrix (TOM) that measured gene connectivity. A dissimilarity matrix (1-TOM) was subsequently produced. To generate gene modules, we used an average linkage hierarchical clustering method with the TOM-based dissimilarity metric to group genes with similar expression profiles. The dendrogram’s gene groupings were subjected to an adjustment, setting the minimum size for each cluster to 30 while the sensitivity was established at 4. Subsequent to the clustering of modules, further analysis was conducted by computing the dissimilarity of module eigengenes. A cut-line was selected for merging the module dendrogram, and some of the modules were merged. A cut-line of 0.2 was selected for merging the modules and a total of 18 co-expression modules were finally obtained. It is worth mentioning that the grey module encompassed a cluster of genes that lacked any definitive assignment to any module (Alderden et al., 2018).

### 2.4 Functional enrichment analysis

The Gene Ontology (GO) resource provides structured computable data regarding the functions of genes and gene products (Consortium, 2019). The GO resource is widely used along with the Kyoto Encyclopedia of Genes and Genomes (KEGG) database for analyzing the functions of genes (Kanehisa and Goto, 2000). In this study, functional enrichment analysis was performed using the clusterProfiler package in R (Yu et al., 2012) and the results were visualized with Sangerbox (Shen et al., 2022). GO and KEGG analyses were performed for identifying the DEGs in OP that overlapped with the most significant module genes of OP, and the intersecting DEGs were defined as the OP set. The genes associated with smoking-related OP that overlapped with the DEGs in COPD were subjected to GO and KEGG enrichment analyses. The intersecting DEGs were subsequently used for screening the candidate genes.

### 2.5 Application of machine learning algorithms

The least absolute shrinkage and selection operator (LASSO) regression method is widely used for selecting and regularizing variables for increasing the predictive accuracy and comprehensibility of statistical models (Yang et al., 2018). The random forest (RF) method provides an effective approach for predicting continuous variables and obtaining reliable forecasts owing to its higher accuracy, sensitivity, specificity, and no limits on variable conditions (Ellis et al., 2014). LASSO regression and RF analyses were performed in this study using the glmnet (Zhang et al., 2019) and randomForest packages, respectively, in R. The genes that were identified by both LASSO and RF were selected as predictor genes for constructing the logistic regression models.

### 2.6 Construction and validation of logistic regression model

A logistic regression model was constructed based on the identified predictor genes using the glmnet package in R (Friedman et al., 2010). The GSE10006 and GSE103174 validation datasets were used for evaluating the accuracy of the model based on the receiver operating characteristic (ROC) curve and the area under the curve (AUC). The results were visualized using the pROC package in R (Robin et al., 2011), and AUCs >0.8 were regarded as ideal.

## 3 Results

### 3.1 Identification of DEGs

**FIGURE 2:**
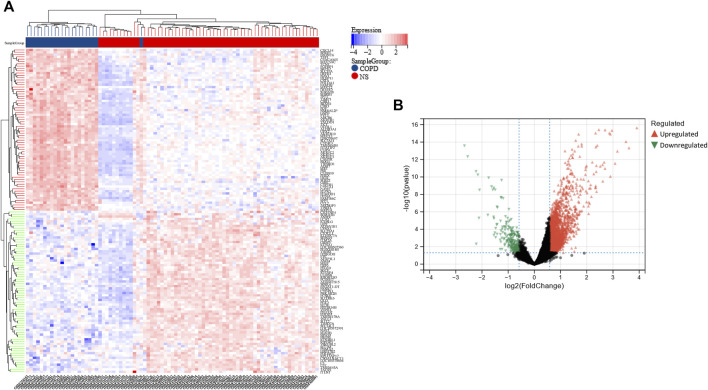
Heatmap and volcano plot of the DEGs in the COPD dataset. **(A)** The rows depict the DEGs, and the columns represent the respective COPD cases or control samples. The upregulated and downregulated DEGs are depicted in red and blue, respectively. **(B)** The upregulated and downregulated DEGs are depicted in red and green, respectively.

**FIGURE 3:**
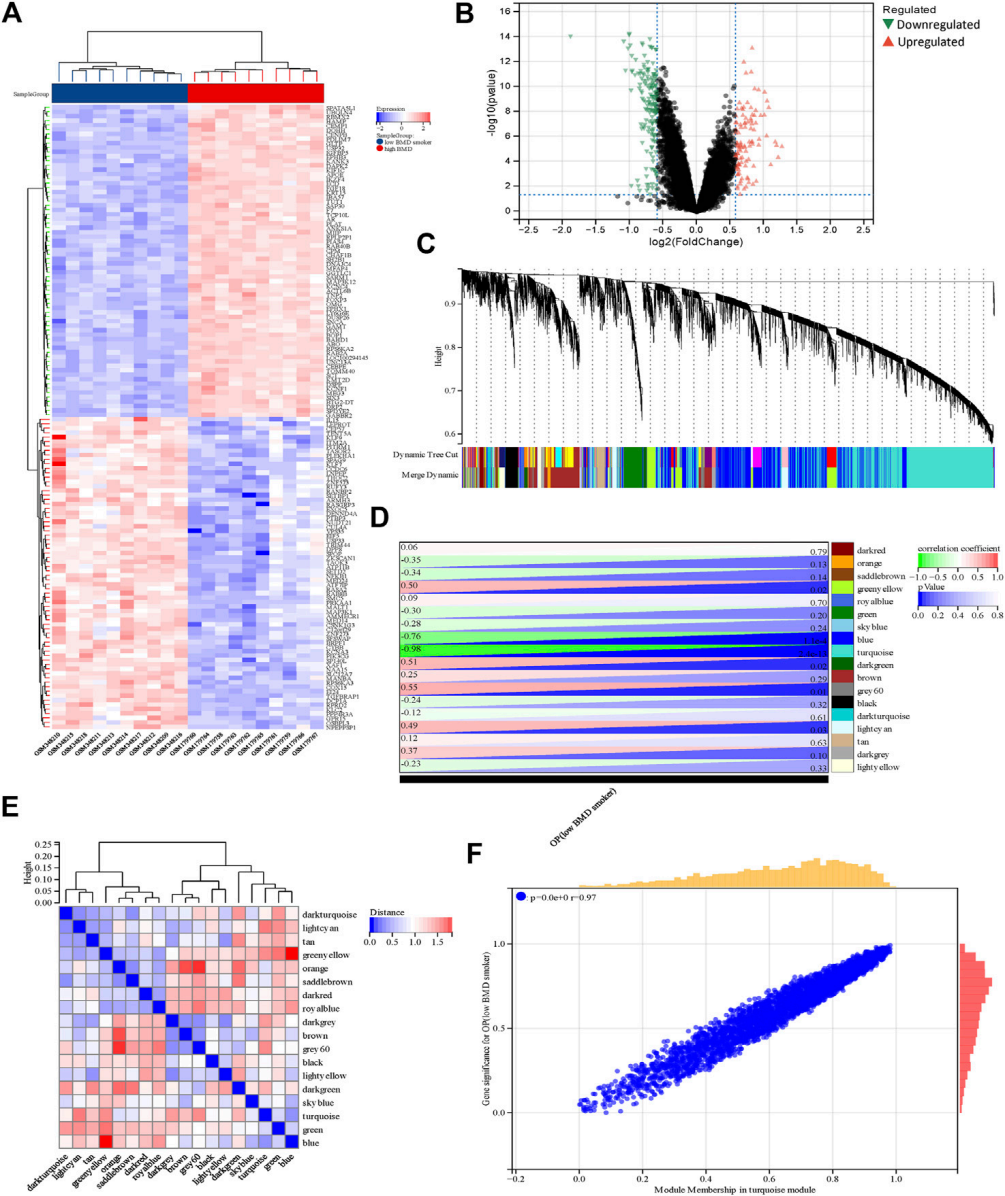
Identification of DEGs and genes associated with smoking-related OP by WGCNA. **(A)** The rows depict the DEGs, and the columns represent the respective smoking-related OP cases or control samples. The upregulated and downregulated DEGs are depicted in red and blue, respectively. **(B)** The upregulated and downregulated DEGs are depicted by red and green triangles, respectively. **(C)** The gene co-expression modules are represented in different colors in the gene tree. **(D)** Heatmap depicting the association between gene modules and OP. The turquoise module was significantly correlated with OP. The numbers on the left and right depict the correlation coefficients and *p*-values, respectively. **(E)** Heatmap depicting the adjacency of the eigengenes. **(F)** Plot depicting the correlation between module membership and gene significance of the DEGs in the turquoise module. OP, osteoporosis metabolic syndrome.

Using the limma package in R, a total of 2,858 DEGs were detected in the COPD dataset. Of these, 2,596 genes were found to be upregulated while 262 were downregulated (Figure 2A, B). Additionally, a total of 280 DEGs were identified in the OP dataset, of which 109 and 171 genes were upregulated and downregulated, respectively (Figure 3A, B).

### 3.2 WGCNA and identification of key modules

The soft threshold power was set at a value of 4, and the scale-free topological index was established at 0.85. The resultant gene tree and corresponding module colors were generated and visually represented using distinct color schemes (Figure 3C, E), in 18 gene modules. The correlation between smoking-related OP and the gene modules is depicted in Figure 3D. The turquoise module, which comprised 982 genes, exhibited the highest correlation with OP (correlation coefficient = −0.98, *p* = 2.4 e-13) and this module was regarded as the pivotal module for the subsequent analyses. The correlation between module membership and gene significance in the turquoise module was determined for elucidating the association between the genes and smoking-related OP. The findings revealed a significant positive correlation between module membership and gene significance (r = 0.97, *p* < 0.0001) as depicted in Figure 3F. The findings showed a significant correlation between the genes within the turquoise module and smoking-related OP.

### 3.3 Functional enrichment analysis of DEGs associated with smoking-related OP

**FIGURE 4:**
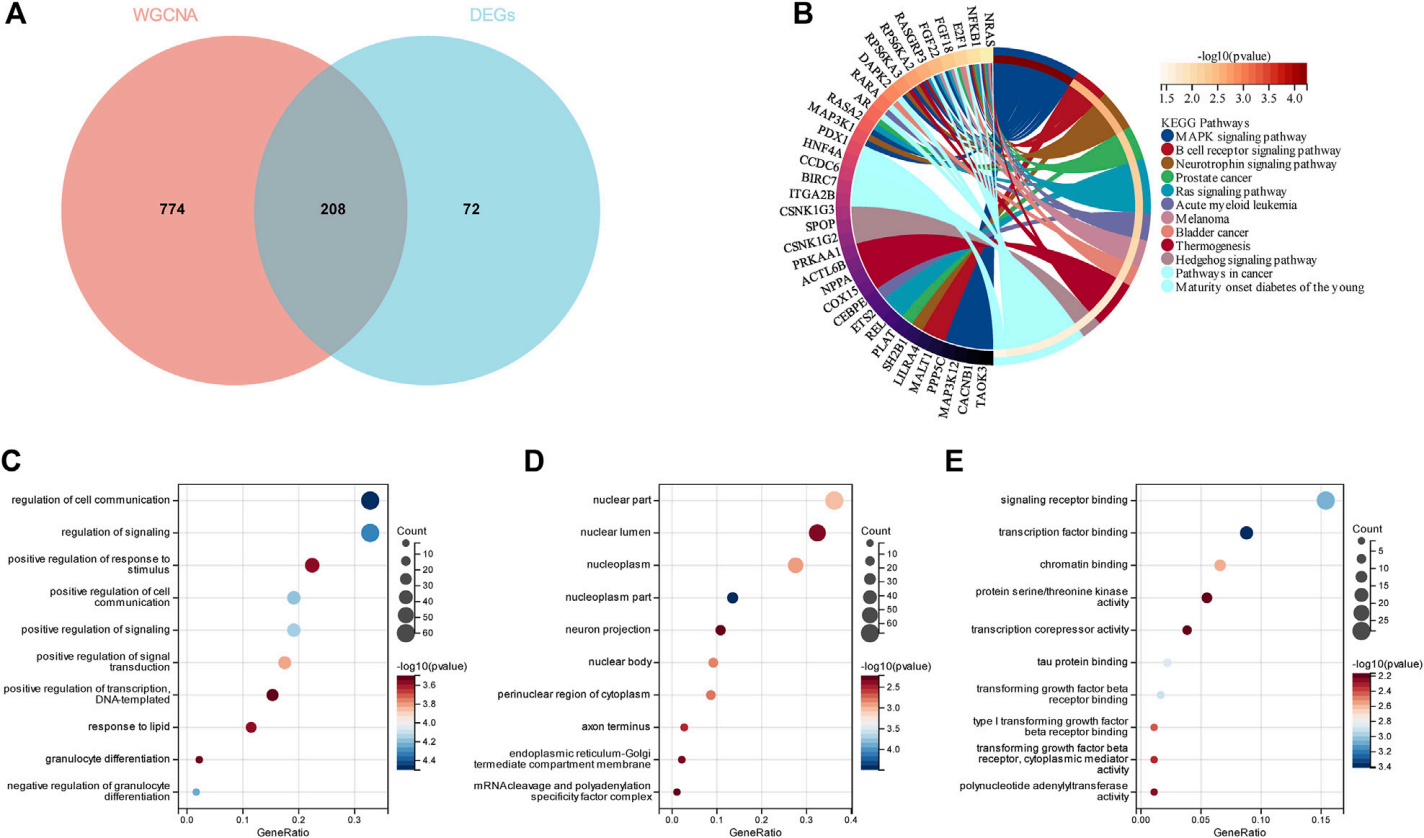
Enrichment analyses of the intersecting genes in smoking-related OP. **(A)** Venn diagram depicting the 208 genes identified from the intersection of 280 DEGs and 982 genes in the turquoise module. **(B)** KEGG pathway analysis of the 208 intersecting genes. The different colors represent the different significantly enriched pathways and related enriched genes. **(C–E)** GO analysis of the intersecting genes that were enriched in different terms in the BP, CC, and MF categories. The *x*-axis represents the ratio of genes that were enriched in the different GO terms and the *y*-axis represents the different GO terms. The diameters of the circles correspond to the number of genes, and the colors represent the *p*-values.

To evaluate the GSE13850 dataset’s reliability in providing insights into the pathogenesis of smoking-related osteoporosis, functional enrichment analysis was conducted on the DEGs that intersected with the genes found within the turquoise module. By intersecting the 280 DEGs with the 982 genes in the turquoise module, a total of 208 genes were identified as shared genes (Figure 4A). The results of GO enrichment analysis revealed that the 208 intersecting genes were primarily enriched in the nucleoplasm term in the cellular component (CC) category of GO; the transcription factor binding, signaling receptor binding, and beta receptor binding terms in the molecular function (MF) category; and the regulation of cell communication and signaling term in the biological process (BP) category (Figure 4C–E). The results of KEGG pathway analysis revealed that the 208 intersecting genes were primarily enriched in the MAPK signaling pathway and B cell receptor signaling pathway terms (Figure 4B). Altogether, the results of enrichment analyses revealed that the 208 genes of OP were primarily related to inflammatory responses, which indicated that the dataset selected herein could provide reliable insights and was therefore used for further analyses.

### 3.4 Enrichment analyses of intersecting DEGs in COPD and OP

**FIGURE 5:**
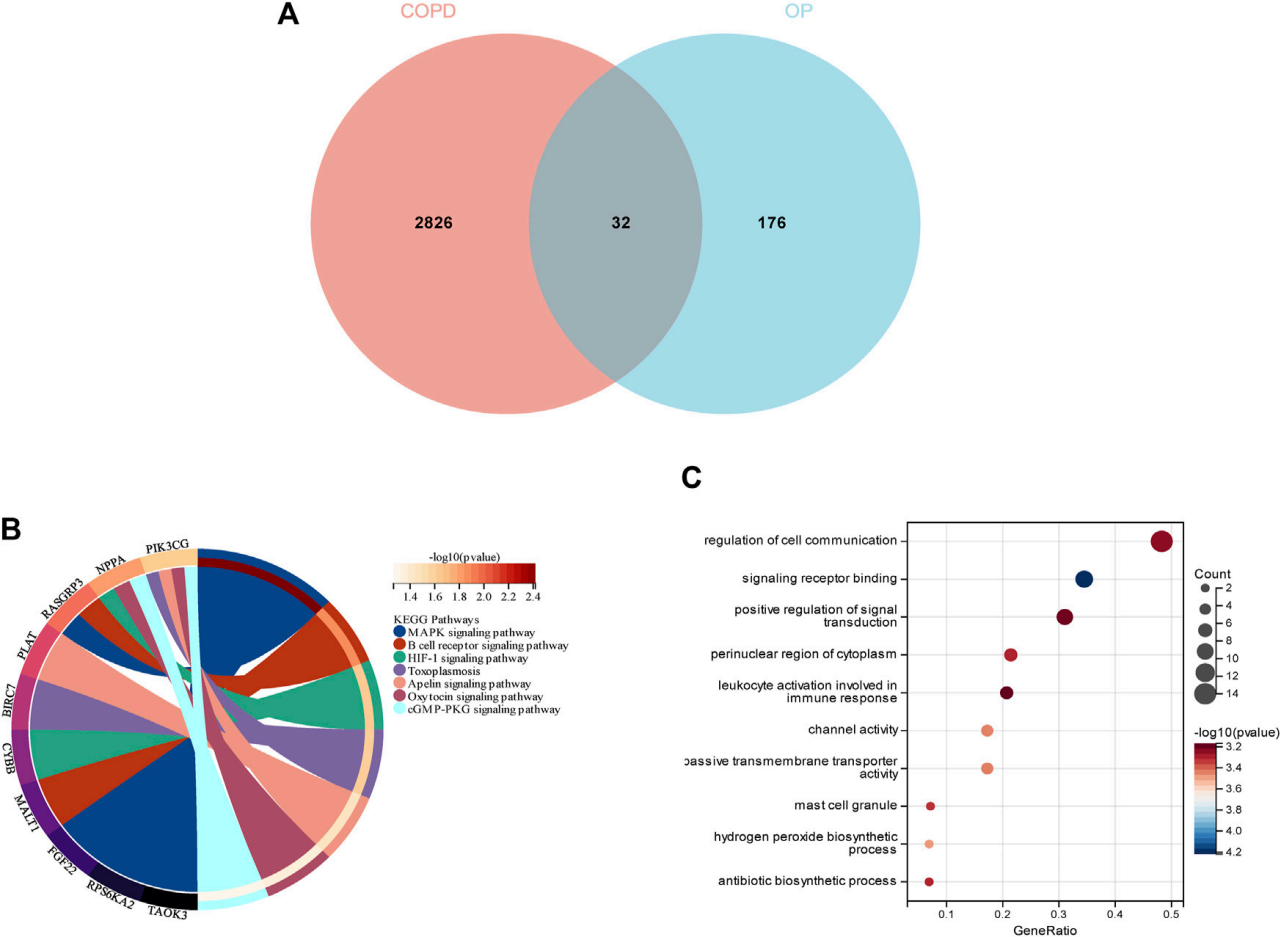
Enrichment analysis of the genes identified from the intersection of DEGs in COPD and smoking-related OP. **(A)** The Venn diagram depicts the 32 genes that were identified from the intersection of the DEGs in COPD and OP. **(B)** Results of KEGG pathway analysis of the 32 intersecting genes. The different significantly enriched pathways and related enriched genes are depicted in different colors. **(C)** Results of GO enrichment analysis of the intersecting genes. The *x*-axis represents the ratio of genes enriched in the different GO terms and *y*-axis represents the different GO terms. The diameters of the circles correspond to the number of genes, and the colors represent the *p*-values.

In order to demonstrate whether smokers with low BMD harbor genes that may be related to the pathogenesis of smoking-related COPD, a total of 32 genes were identified as the intersection of DEGs between COPD and OP, and visualized using a Venn diagram (Figure 5A). The results of KEGG enrichment analysis (Figure 5B) revealed that the 32 intersecting genes were primarily enriched in the MAPK signaling pathway, B cell receptor signaling pathway, HIF-1 signaling pathway, toxoplasmosis, apelin signaling pathway, oxytocin signaling pathway, and cGMP-PKG signaling pathway. The results of GO enrichment analysis (Figure 5C) revealed that the 32 intersecting genes were enriched in the biosynthetic process and regulation of signaling terms in the BP category, mast cell granule and apical part of cell terms in the CC category, and signaling receptor binding and channel activity terms in the MF category.

### 3.5 Identification of candidate genes by machine learning algorithms

**FIGURE 6:**
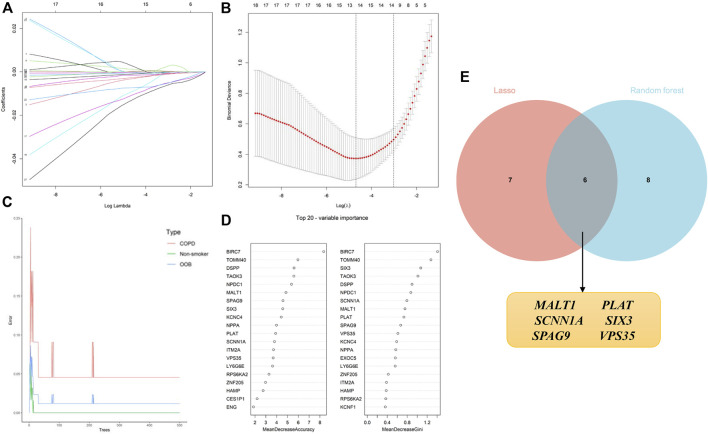
Application of machine learning algorithms for screening candidate diagnostic biomarkers in smokers with COPD and low BMD. **(A, B)** Screening of biomarkers using the LASSO model. The number of genes (*n* = 13) corresponding to the lowest point of the curve was most suitable. **(C, D)** The error in the COPD group, as identified by the RF algorithm; the control group and genes are ranked based on the importance score. **(E)** Venn diagram depicting the six candidate diagnostic genes identified using the LASSO and RF machine learning algorithms.

The identification of candidate genes was performed by conducting logistic regression analysis with the aid of LASSO and RF machine learning algorithms. A total of 13 candidate biomarkers were identified using the LASSO regression algorithm (Figure 6A, B), and the genes were ranked using the RF algorithm by calculating the importance of each gene (Figure 6C,D). The 14 genes predicted using the RF algorithm and the 13 candidate genes identified using the LASSO algorithm were overlapped, and the intersecting genes were visualized using a Venn diagram (Figure 6E). A total of six intersecting genes, namely, MALT1, PLAT, SCNN1A, SIX3, SPAG9, and VPS35, were identified for final validation.

### 3.6 Construction of logistic regression model and analysis of ROC curve

**FIGURE 7:**
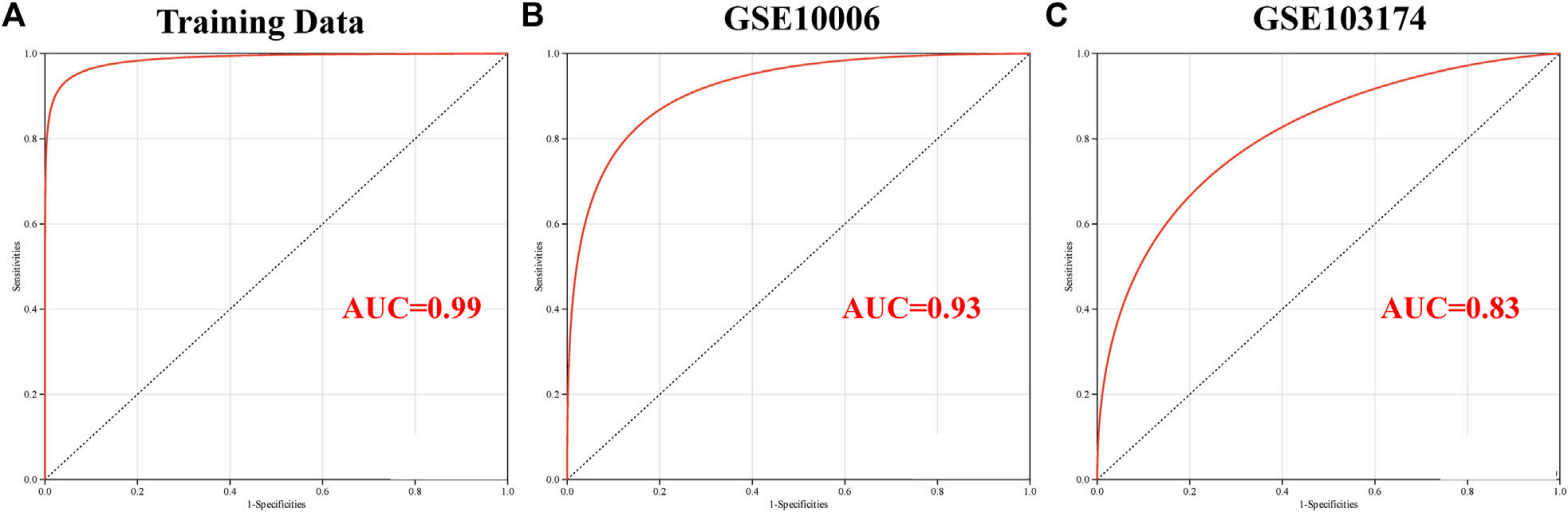
Validation of the key genes involved in crosstalk using the independent external datasets. Analysis of the ROC curve of the key genes involved in crosstalk using the **(A)** GSE11784, **(B)** GSE10006, and **(C)** GSE103174 datasets.

A logistic regression model was constructed based on the six candidate genes, namely, MALT1, PLAT, SCNN1A, SIX3, SPAG9, and VPS35, using a logistic regression algorithm. The results demonstrated that the predictive model constructed using these six genes had superior diagnostic performance, with an AUC of 0.99 with the training set (Figure 7A). The results of ROC curve analysis revealed that the model achieved reliable prediction results with the GSE10006 and GSE103174 external datasets (Figure 7B, C), with AUCs of 0.93 and 0.83, respectively, and the findings were consistent with the results obtained using the training set. The findings revealed that the six candidate genes might serve as potential biomarkers of COPD and smoking-related OP; however, further experimental studies are necessary for validating these findings.

### 3.7 Analysis of immune cell infiltration

**FIGURE 8:**
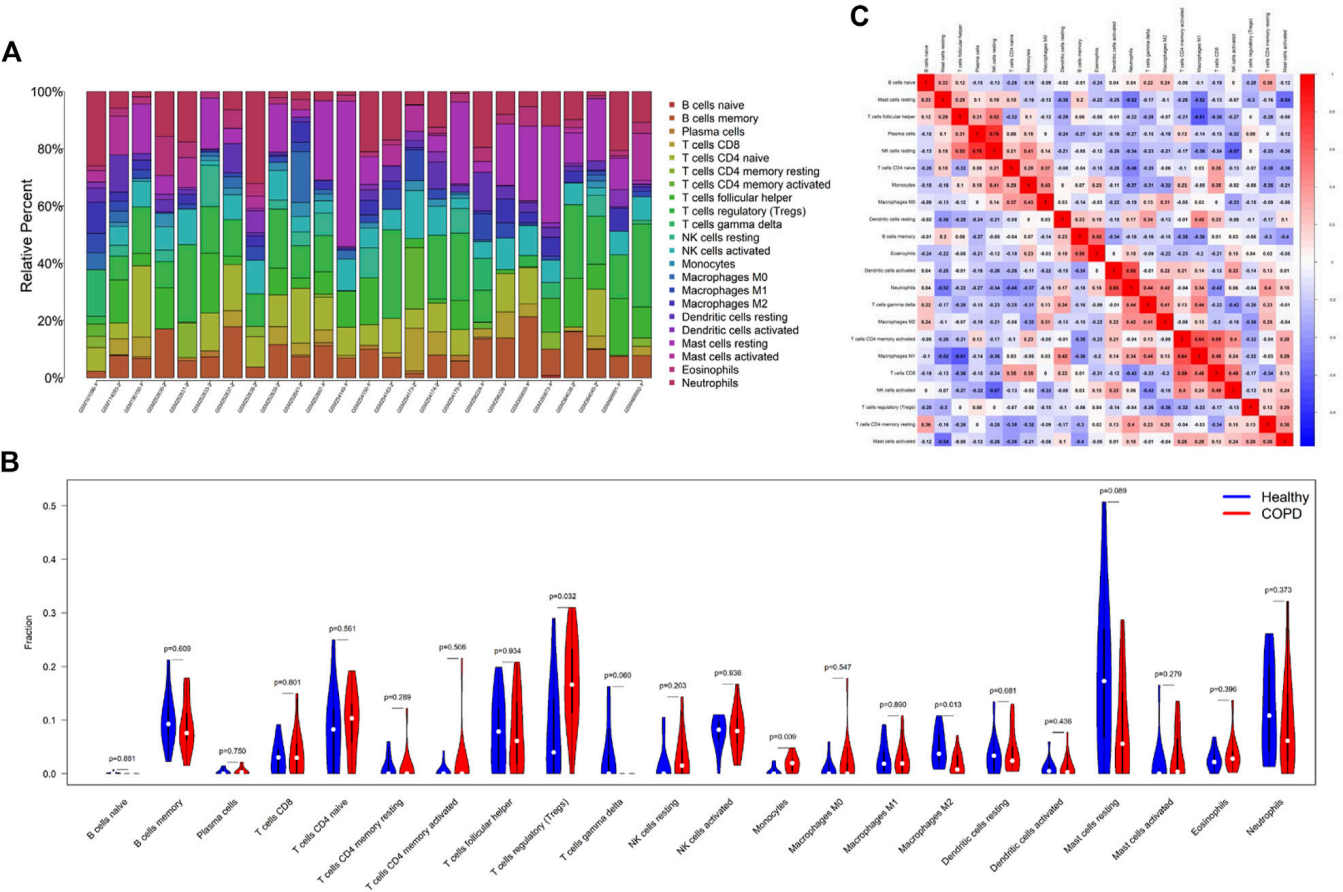
Comparison of immune cell infiltration in the COPD and control samples. **(A)** The proportions of 22 types of immune cells in the different samples were visualized using a bar plot. **(B)** Comparison of the proportions of 22 types of immune cells between the COPD and control groups. **(C)** Correlation among the compositions of the 22 types of immune cells. *p < 0.05, **p < 0.01, ***p < 0.001. The horizontal and vertical axes depict the immune cell subtypes.

As the DEGs in COPD and OP were enriched in immune regulation terms, the investigation of immune cell infiltration may offer a more comprehensive understanding of the mechanisms involved in regulating immune responses in COPD. The proportions of 22 types of immune cells identified in the GSE11784 dataset were graphically represented by a bar plot (Figure 8A). The findings revealed that patients with smoking-related COPD had a higher population of regulatory T cells (Tregs) and monocytes, but a lower proportion of M2 macrophages (Figure 8B). By analyzing the correlation among the 22 different types of immune cells (Figure 8C), it was observed that the fraction of plasma cells exhibited a positive correlation with the fraction of resting NK cells (r = 0.76), while the fraction of resting dendritic cells was positively correlated with the neutrophil fraction (r = 0.64). The fraction of M1 macrophages was negatively correlated to the fraction of resting mast cells (r = −0.52) and follicular helper T cells (r = −0.61), but positively correlated to the fraction of memory-activated CD4 T cells (r = 0.64).

## 4 Discussion

There has been a growing public consciousness regarding the detrimental impact of cigarette smoke exposure. Despite significant advancements in regulating tobacco usage, cigarette smoking remains a formidable global public health concern, with no clear end in sight. (Petzold et al., 2009; Alberg et al., 2014). Smoking-induced genetic alterations influence the secretion of hormones and bone metabolism in the elderly population, which regulate the pathogenesis of OP (Yoon et al., 2012; Marom-Haham and Shulman, 2016). Cigarette smoking is widely acknowledged as a major contributing factor to the development of COPD, with estimates suggesting that approximately 20%–25% of smokers eventually develop this condition (Løkke et al., 2006). Despite numerous studies on COPD in the past few decades, the mechanisms underlying the pathogenesis of COPD remains poorly understood. The precise effect of cigarette smoking on the pathogenesis of smoking-related OP and COPD, and further investigation is warranted to elucidate the potential correlation between these disorders. A growing body of evidence suggests that cigarette smoking-induced airway inflammation is linked to dysregulated immune cell infiltration in patients diagnosed with COPD (Cruz et al., 2019; Bu et al., 2020). It is worth noting that smoking has the potential to elicit detrimental alterations in the immune system, leading to diseases that stem from aberrant regulation of immune cells (Wang et al., 2021; Luan and Si, 2022). Therefore, the identification of effective and novel diagnostic biomarkers of smoking-related OP and COPD within immune cell components represents a promising domain of investigation, with potential implications for early intervention and improved clinical outcomes.

So far, however, no study has investigated the combined effects of smoking-related OP and COPD. There are no studies on the application of machine learning methods or the construction of logistic regression models for predicting the shared gene signatures of smoking-related OP and COPD. The present study performed integrated bioinformatics analyses and applied machine learning algorithms for identifying potential biomarkers and evaluating the diagnostic value of smoking-related OP in patients with COPD. Notably, the study identified six immune-associated candidate diagnostic genes, namely, MALT1, PLAT, SCNN1A, SIX3, SPAG9, and VPS35, that could serve as biomarkers of OP in patients with COPD.

The MALT1 protein exhibits expression and functional activity within osteoclasts, and is stimulated by receptor activator of NF-κB ligand (RANKL) in preosteoclasts. Previous investigations have demonstrated that individuals with combined immunodeficiency attributed to MALT1 deficiency present with diminished bone mineral density, leading to the occurrence of several low-impact fractures. Monajemi et al. demonstrated that Malt1 deficient mice develop an OP phenotype that is characterized by increased osteoclastogenesis *in vivo*, and is primarily caused by the inflammation of osteoclasts (Monajemi et al., 2019). Furthermore, MALT1 is of paramount importance in regulating innate and adaptive immune signaling by adjusting the activation threshold of immune cells. In a murine model of autoimmune arthritis, it was previously reported that the deletion of MALT1 in T cells resulted in the development of spontaneous OP, which was concomitant with changes in the frequency of dysfunctional Treg cells (Gilis et al., 2019). The CARMA1-BCL10-MALT1 (CBM) complex is known to contribute to the pathogenesis and progression of allergic inflammation and diseases, including COPD (DeVore and Khurana Hershey, 2022). PLAT has been identified as an immune checkpoint gene that exerts a critical influence on the prognosis of various cancers, such as breast cancer and hepatocellular carcinoma, by modulating the levels of immune molecules and regulating the infiltration of immune cells in the tumor microenvironment (Wang et al., 2021; Hu et al., 2022). Using gene network analysis, a prior study identified PLAT as a SARS-CoV-2 target and elucidated its potential involvement in COVID-19’s inflammatory response, metabolism of reactive oxygen species, and immune response. (Saik and Klimontov, 2022). Wan et al. conducted a large-scale genome-wide analysis of two COPD cohorts and obtained DNA methylation data for 27,578 CpG sites in 14,475 genes. The study revealed that the systemic use of steroids is associated with differential site-specific methylation of several hub genes, including SCNN1A, which partakes in various biological processes, including cellular ion homeostasis and the activation of leukocytes and lymphocytes (Wan et al., 2012). A separate investigation has provided evidence that SCNN1A is associated with unfavorable clinical outcomes in individuals afflicted with ovarian cancer, and exerts an impact on the immune cell infiltration patterns within the tumor microenvironment (Lou et al., 2022). A previous study reported that the SIX3 homeodomain transcription factor was dysregulated in a TNF-α-stimulated inflammatory model of epithelial cells, and SIX3 was found to affect cellular apoptosis and motility under inflammatory conditions (Korthagen et al., 2015). Additionally, Zheng et al. revealed the critical role of SIX3 loss-of-function in breast cancer progression, tumorigenesis, and metastasis. (Zheng et al., 2018). SPAG9 is a newly identified member of cancer/testis antigens and has been found to induce a specific immune response in several patients with cancer. SPAG9 promotes the growth and metastasis of epithelial cells by regulating the c-Jun N-terminal kinase (JNK) and mitogen-activated protein kinase (MAPK) signaling pathways (Pan et al., 2018). The MAPK signaling pathway is a crucial mediator in the pathogenesis of OP and COPD, and has been reported to regulate the activity of osteoblasts and osteoclasts (Malakoti et al., 2022) and modulate airway inflammation and remodeling (Mei et al., 2022). Xia et al. reported that VPS35 partakes in regulating the trafficking, signaling, and functions of receptor activator of nuclear factor kappaB ligand (RANK) (Xia et al., 2013). The loss function of VPS35 alters the RANKL-induced distribution of RANK, enhances the sensitivity of RANKL, sustains RANKL signaling, and induces the formation of hyper-resorptive osteoclasts. Another study demonstrated that VPS35 can be potentially applied for the diagnosis OP and the VPS35 gene was found to play a potential role in the pathogenesis of OP (Xia et al., 2017).

Immunological and inflammatory modulation has been observed throughout all stages of COPD as reported in previous studies. The present study revealed an elevated frequency of Treg cells and monocytes and a reduced frequency of M2 macrophages in COPD patients, in agreement with previous investigations. Comprehending the underlying mechanisms that underlie inflammatory signaling can furnish valuable insights towards the development of diagnostic methodologies (Chen et al., 2022) and facilitate the identification of innovative therapeutic agents for COPD related to smoking.

The present study has certain limitations with are described hereafter. Firstly, the other related genes could not be identified in this study owing to the limited number of studies on the genetic variations induced by smoking, and the fact that only the GSE13850 dataset of GEO contained data regarding smoking-related OP. Secondly, as only a limited number of clinical samples could be included in our study, the findings obtained herein need to be confirmed using a larger cohort. Thirdly, although the six identified genes were primarily involved in regulating the inflammatory and immune pathways, further in-depth molecular biology experiments and prospective clinical trial cohorts need to be designed for validating the mechanism of action of these candidate genes.

Based on the results of this study, there are several future directions that can be explored. Firstly, further validation of the identified feature genes or exploration of other feature genes can be conducted in larger sample sizes to improve the accuracy and reliability of the results. Additionally, functional analysis can be performed on these feature genes to better understand their biological roles in COPD and smoking-related OP. Furthermore, biological experiments can be used to explore the mechanisms of action of these identified feature genes and further investigate the effectiveness and safety of targeting these genes for the treatment of these diseases. In summary, this study provides valuable insights into potential biomarkers and therapeutic targets for COPD and smoking-related OP. Further research and development in these areas may lead to progress in the diagnosis, treatment, and management of these diseases.

## 5 Conclusion

The present study systematically identified six candidate diagnostic genes, namely, MALT1, PLAT, SCNN1A, SIX3, SPAG9, and VPS35, which were the shared gene signatures of smoking-related OP and COPD, through a combined approach of integrated bioinformatics analyses and machine learning algorithms. The findings revealed that the association between smoking-related OP and COPD and the influence of cigarette smoking on the pathogenesis of these two diseases could be closely related to the infiltration of immune cells. The aforementioned genes and immune cells hold promise as potential targets for immunotherapeutic intervention in individuals afflicted with smoking-related OP and COPD.

## Data Availability

The original contributions presented in the study are included in the article/Supplementary Material, further inquiries can be directed to the corresponding authors.
